# Prediction of lncRNA-protein interactions using HeteSim scores based on heterogeneous networks

**DOI:** 10.1038/s41598-017-03986-1

**Published:** 2017-06-16

**Authors:** Yun Xiao, Jingpu Zhang, Lei Deng

**Affiliations:** 1 School of Software, Central South University, Changsha, 410075 China; 2 School of Information Science and Engineering, Central South University, Changsha, 410083 China; 3 Shanghai Key Laboratory of Intelligent Information Processing, Shanghai, 200433 China

## Abstract

Massive studies have indicated that long non-coding RNAs (lncRNAs) are critical for the regulation of cellular biological processes by binding with RNA-related proteins. However, only a few experimentally supported lncRNA-protein associations have been reported. Existing network-based methods are typically focused on intrinsic features of lncRNA and protein but ignore the information implicit in the topologies of biological networks associated with lncRNAs. Considering the limitations in previous methods, we propose PLPIHS, an effective computational method for Predicting lncRNA-Protein Interactions using HeteSim Scores. PLPIHS uses the HeteSim measure to calculate the relatedness score for each lncRNA-protein pair in the heterogeneous network, which consists of lncRNA-lncRNA similarity network, lncRNA-protein association network and protein-protein interaction network. An SVM classifier to predict lncRNA-protein interactions is built with the HeteSim scores. The results show that PLPIHS performs significantly better than the existing state-of-the-art approaches and achieves an AUC score of 0.97 in the leave-one-out validation test. We also compare the performances of networks with different connectivity density and find that PLPIHS performs well across all the networks. Furthermore, we use the proposed method to identify the related proteins for lncRNA MALAT1. Highly-ranked proteins are verified by the biological studies and demonstrate the effectiveness of our method.

## Introduction

Long non-coding RNAs (lncRNAs) are becoming critically important for the understanding of life sciences. Studies have indicated that lncRNAs play critical roles in many important biological processes such as chromatin modification^1^, transcriptional and post-transcriptional regulation^2, 3^, and human diseases^4, 5^. Relating proteins with Long non-coding RNAs (lncRNAs) is a tremendous and meaningful task in human health with applications in understanding lncRNA mechanisms, diagnosis and therapy^6, 7^. In general, lncRNAs exert functions by interfacing with corresponding RNA-binding proteins. Thus, identifying lncRNA interacted proteins is significant to understand complex functions of lncRNA and molecular mechanism^8, 9^ of disease progression and cellular circuitry^10, 11^.

Since experimental methods to detect lncRNA-protein interactions are time-consuming and costly, several computational approaches have been reported for predicting RNA-binding proteins (RBPs). For example, Pancaldi *et al*.^12^ proposed a method to predict ncRNA-protein interactions in 2011 and a approach named RPISeq was presented by Muppirala *et al*.^13^ at the same year, which was constructed by using the features derived from protein and RNA sequences. They trained Random Forest (RF) and Support Vector Machine (SVM) classifiers using 3-mer and 4-mer conjoint triad features for amino acid and nucleotide sequences, respectively^14^. Bellucci *et al*. created a method named catRAPID^15^ by exploiting the physicochemical properties including secondary structure, hydrogen bonding and van der Waals propensities. Wang *et al*. proposed an approach based on Naíve Bayes (NB) and Extended NB (ENB) classifiers using the similar data and features supported in Muppirala *et al*.’s work^16^. In 2013, IncPro^17^ was created by Lu *et al*. using three types of classical protein secondary structures, hydrogen-bond and Van der Waals propensities, and six types of RNA secondary structures (RSS).

Nevertheless, all of these methods only focus on intrinsic features of lncRNA and protein but ignore the information implicit in the topologies of biological networks associated with lncRNAs. On the other hand, biological network-based methods was already widely used in many types of studies, such as disease gene prioritization^18^ and drug-target interaction prediction and some of them have achieved good performances. One of the most commonly used approach is guilt-by-association (GBA)^19^, which provides the central top-down principle for analyzing gene networks in functional terms or assessing their quality in encoding functional information. New emerged methods, including the Katz method^20^, Combining dATa Across species using Positive-Unlabeled Learning Techniques(CATAPULT)^19^, Random Walk with Restart (RWR)^21^, and LncRNA-protein Interaction prediction based on Heterogeneous Network model (LPIHN)^22^, have extended the association from just direct protein interactions to more distant connections in various ways. The KATZ measure^20^ is a weighted sum of the number of paths in the network that measures the similarity of two nodes. CATAPULT^19^ is a supervised machine learning method that uses a biased support vector machine where the features are derived from walks in a heterogeneous gene-trait network. RWR^21^ is a method for prioritization of candidate genes by use of a global network distance measure, random walk analysis, for definition of similarities in protein-protein interaction networks and it add weight to the assumption that phenotypically similar diseases are associated with disturbances of subnetworks within the larger protein interactome that extend beyond the disease proteins themselves. LPIHN^22^ is a network-based method by implement a random walk on a heterogeneous network. PRINCE is a global method based on formulating constraints on the prioritization function that relate to its smoothness over the network and usage of prior information. Compared with LPIHN and RWR, PRINCE propagates information in a smaller network but contains more connotative meaning when build the initial probability values and has made great performance in gene prioritization^23^ and disease identification^24^.

However, many existing network-based methods simply view objects in heterogeneous networks as the same type and do not consider the subtle semantic meanings of different paths. In this paper, we adopt a method named HeteSim, which is a path-based measure to calculate the relevance between objects in heterogeneous network^25^. The basic idea is that similar objects are more likely to be related to some other objects. Considering the relatedness of heterogeneous objects is path-constrained, HeteSim gives a uniform and symmetric measure for arbitrary paths to evaluate the relatedness of heterogeneous object pair (same or different types) with one single score. Due to the relevance path not only captures the semantics information but also constrains the walk path, the score is also a path-based similarity measure.

**Figure 1:**
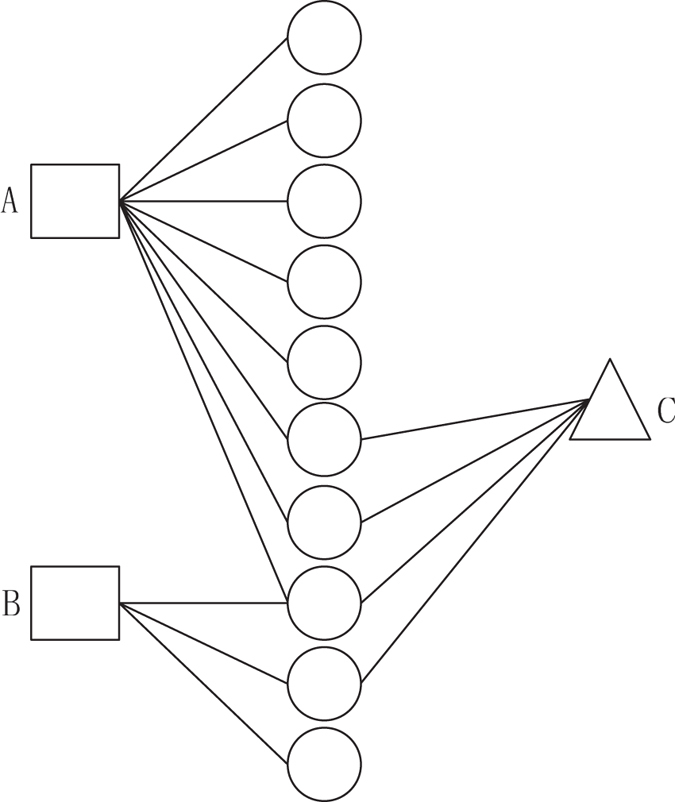
Example of understanding HeteSim measure. Circle, squares and triangle denote three different kinds of objects in the heterogeneous network.

An example of HeteSim score is illustrated in (Fig. 1). The number of paths from A to C and B to C is 3 and 2, respectively. The walk count between A and C is larger than B and C, which might indicate that A is more closer to C than B. But the connectivity between B and C is more intense than A and C in the sight of HeteSim score, since most edges starting from B are connected with C, when A only has a small part of edges connected with C.

**Figure 2:**
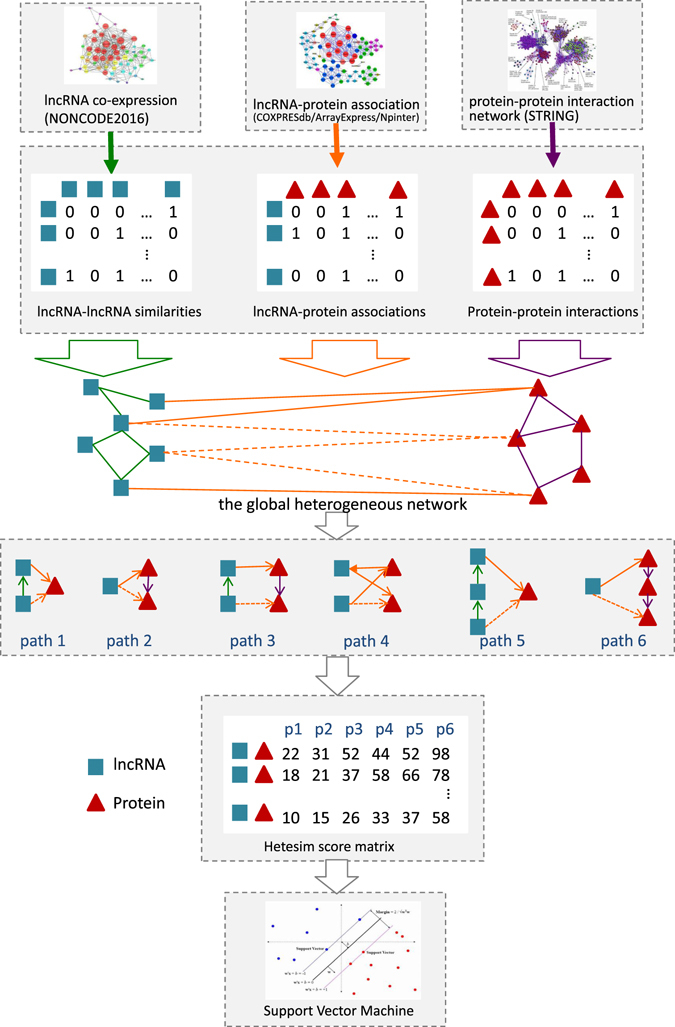
Flowchart of PLPIHS. It includes three steps: (1) constructing a heterogeneous network consisting of a lncRNA-lncRNA similarity network, a lncRNA-protein association network and a protein-protein interaction network; (2) using the HeteSim measure to calculate a score for each lncRNA-protein pair in each path; (3) combining the scores of different paths with a SVM classifier and making predictions.

Here, we propose a method named PLPIHS (Fig. 2) to predict lncRNA-Protein interactions using HeteSim scores. We first construct a heterogeneous network consisting of a lncRNA-lncRNA similarity network, a lncRNA-protein association network and a protein-protein interaction network. Then, we use the HeteSim measure to calculate the score for each lncRNA-protein pair in the network. A SVM classifier is built based on the scores of different paths. We compare our PLPIHS with PRINCE, RWR and LPIHN and find that PLPIHS outperforms the other methods in many performance measures.

## Results

### Validation measures

LOOCV(Leave-One-Out Cross Validation)^26^ is implemented on the verified lncRNA-protein associations to evaluate the performance of LPIHN^22^. We leave a known lncRNA-protein pair in turn as the test sample and all the other known lncRNA-protein pairs are regarded as training samples. In order to improve the accuracy of PLPIHS, we remove all connected lncRNAs and proteins while in each validation round. Receiver Operating Characteristic(ROC) curve^27^ is used to evaluate the prediction performance, which plots true-positive rate (TPR, sensitivity or recall) versus false-positive rate (FPR, 1-specificity) at different rank cutoffs. When varying the rank cutoffs of successful prediction, we can obtain the corresponding TPR and FPR. In this way, ROC curve is drawn and the area under the curve(AUC) is calculated as well. For a rank threshold, sensitivity(SEN)^28^ and specificity(SPE)^29^ are defined as follow:

$$\begin{array}{rcl}SEN & = & \frac{TP}{TP+FN}\\ SPE & = & \frac{TN}{TN+FP}\end{array}$$

TN and TP represent the number of negative sites and positive sites that are correctly predicted. FP and FN represent the number of positive sites and negative sites that are wrongly predicted. Meanwhile, some common used measurements, namely, accuracy(ACC), precision(PRE), Mathew correlation coefficient(MCC) and F1-Score^30^, are calculated as follows:

$$\begin{array}{rcl}ACC & = & \frac{TP+TN}{TP+TN+FP+FN}\\ PRE & = & \frac{TP}{TP+FP}\\ MCC & = & \frac{TP\ast TN-FP\ast FN}{\sqrt{(TP+FN)\ast (TP+FP)\ast (TN+FN)\ast (TN+FP)}}\\ F1-Score & = & \frac{2\ast PRE\ast SEN}{PRE+SEN}\end{array}$$

These measurements are also used to assess the capability of PLPIHS during the preprocessing procedure.

### Affection of network preprocessing characteristics

**Figure 3:**
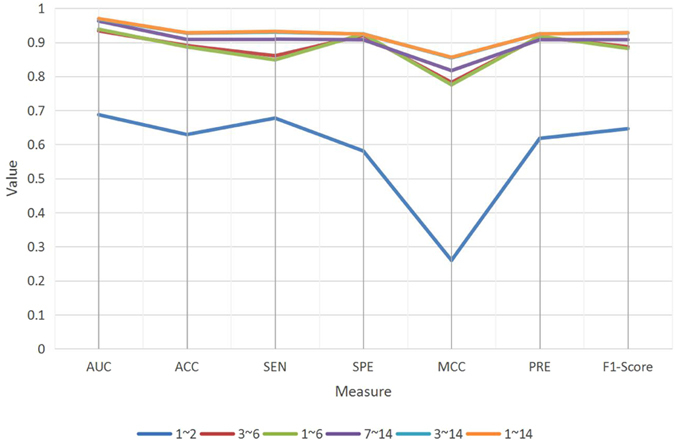
The performance comparison of different paths. The X-axis represents different performance measures and the Y-axis indicates the values of these measures. The colored lines denote the performances of different paths.

In this paper, we only have two kinds of objects, lncRNA and protein. Thus, the paths from a lncRNA to a protein in our heterogeneous network with length less than six is listed in Table 1. In order to pick out the most efficient paths, we compared the performances of these 14 paths under different combinations (Fig. 3). We can see that all paths achieve a favorable status except path 1′~2′. Path 1′~14′ obtains the best performance across all measures, which means that the path with length greater than three contains more significant meanings.

**Table 1:** The paths from a lncRNA to a protein in our heterogeneous network with length less than six.

id	name	path
1	LLP	lncRNA-lncRNA-protein
2	LPP	lncRNA-protein-protein
3	LLPP	lncRNA-lncRNA-protein-protein
4	LPLP	lncRNA-protein-lncRNA-protein
5	LLLP	lncRNA-lncRNA-lncRNA-protein
6	LPPP	lncRNA-protein-protein-protein
7	LPPPP	lncRNA-protein-protein-protein-protein
8	LPPLP	lncRNA-protein-protein-lncRNA-protein
9	LPLPP	lncRNA-protein-lncRNA-protein-protein
10	LPLLP	lncRNA-protein-lncRNA-lncRNA-protein
11	LLPPP	lncRNA-lncRNA-protein-protein-protein
12	LLPLP	lncRNA-lncRNA-protein-lncRNA-protein
13	LLLPP	lncRNA-lncRNA-lncRNA-protein-protein
14	LLLLP	lncRNA-lncRNA-lncRNA-lncRNA-protein

The constant factor *β* is used to control the influence of longer paths. The longer the path length is, the smaller the inhibiting factor is. Path length equals 3 matches with constant *β*, path length equals 4 matches with constant β*β and path length equals 5 matches with constant β*β*β. Table 2 shows that *β* has tiny impact on the final results and *β* = 0.2, 0.4 and 0.7 achieved the best AUC score and the others are not far behind yet.

**Table 2:** The AUC under different beta values.

*β*	0.1	0.2	0.3	0.4	0.5	0.6	0.7	0.8	0.9
AUC	0.969708	0.96971	0.969709	0.96971	0.969709	0.969709	0.96971	0.969708	0.969711

### Performance comparison of networks with different connectivity density

**Figure 4:**
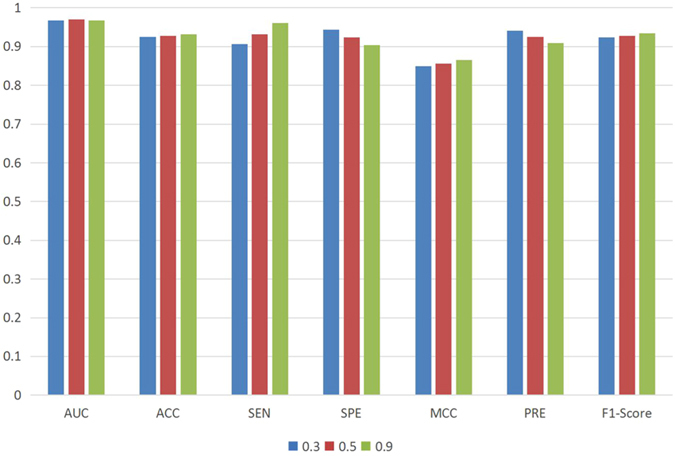
The ROC curves of PLPIHS method under three different levels of sparse networks.

To further verify the dependability of our method, we compare the three networks of different connectivity density under different cutoff value 0.3, 0.5 and 0.9 (see lncRNA-Protein associations). The results are shown in Fig. 4. There are tiny performance differences between different sparse networks. The AUC score of the 0.5 network is higher than that of others while the 0.9 network outperforms others in ACC, SEN, MCC and F1-Score. This suggests that PLPIHS performs well across networks with different densities.

### Comparison with existing network-based methods

To evaluate the performance of the proposed PLPIHS, we compare it with three state-of-the-art methods: PRINCE, LPIHN and RWR. The parameters of PRINCE is set as follow: *α* = 0.9, c = −15, d = log(9999) and the number of propagation iteration is ten. The parameters of LPIHN are consistent with the original implementation as well (*γ* = 0.5, *β* = 0.5 and *δ* = 0.3). For the RWR method, there is only one restart probability r and it’s effects is very slight, which is proved by experiments. The parameter r is set as 0.5 in this comparison.

**Figure 5:**
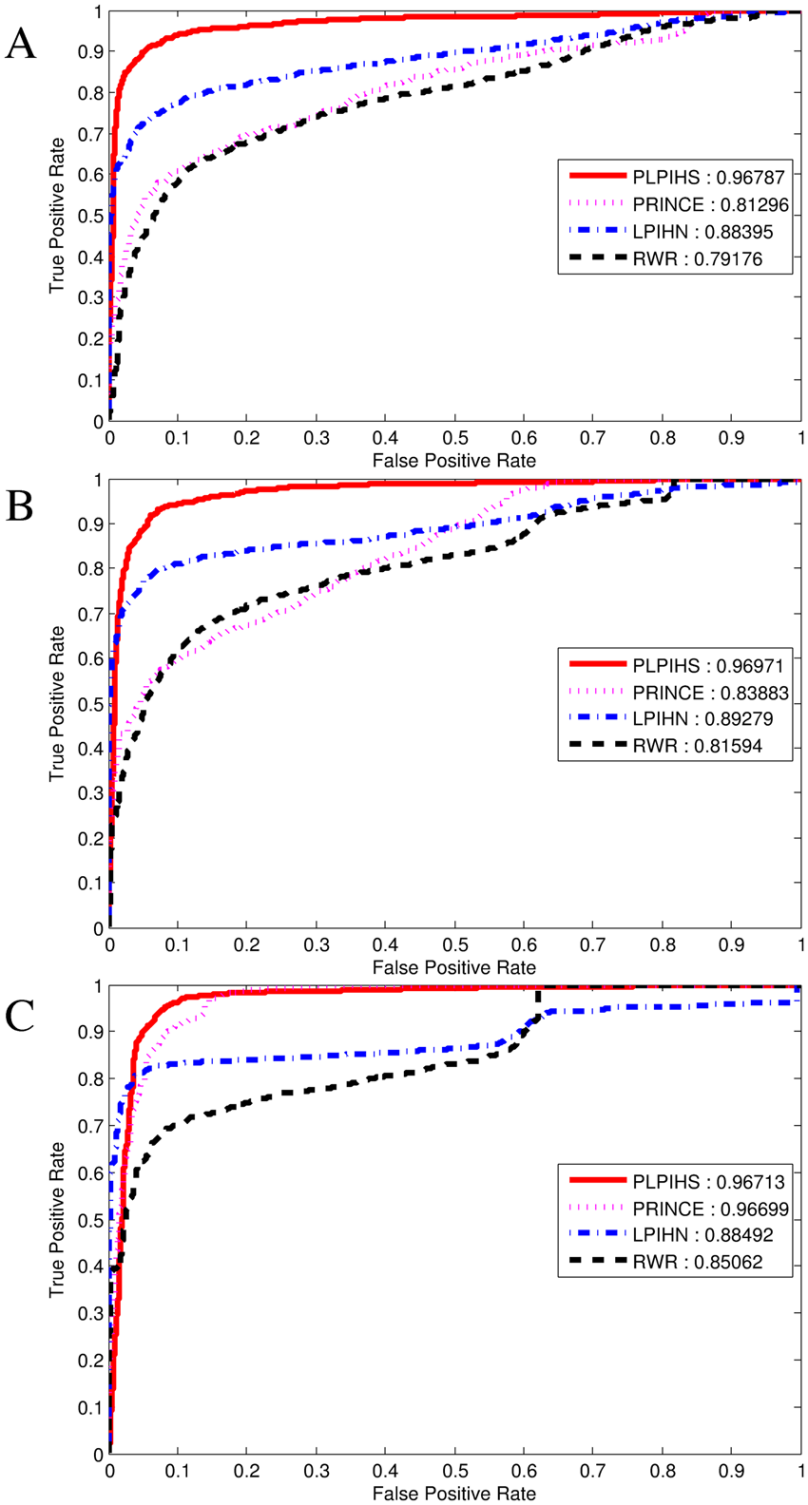
The ROC curves of all methods under 0.3 network, 0.5 network and 0.9 network.

In order to calculate the performance of the different methods, we use a leave-one-out cross validation procedure. We extract 2000 lncRNA-protein associations from the 0.9 network as positive samples, the same number of negative samples are chosen randomly from the 0.3 network as well, avoiding the error caused by imbalance dataset. The gold set which containing 185 lncRNA-protein interactions downloaded from NPinter database has been included in positive pairs as well. In the lncRNA protein prioritization, each lncRNA-protein interaction is utilized as the test set in turn and the remaining associations are used as training data. The whole experiment will be repeated 4000 times to testing each lncRNA-protein pairs in the dataset. ROC curve is drawn based on true positive rate (TPR) and false positive rate (FPR) at different thresholds. The AUC score is utilized to measure the performance. AUC = 1 demonstrates the perfect performance and AUC = 0.5 demonstrates the random performance.The ROC curve of PLPIHS, LPIHS, PRINCE and RWR are plotted in Fig. 5. The results show that the AUC score of PLPIHS in 0.3 network is 96.8%, which is higher than that of PRINCE, LPIHN and RWR, achieving an AUC value of 81.3%, 88.4% and 79.2%, respectively. Similarly, PLPIHS outperforms other methods in 0.5 network and 0.9 network as well.

### Performance evaluation by independent test

**Figure 6:**
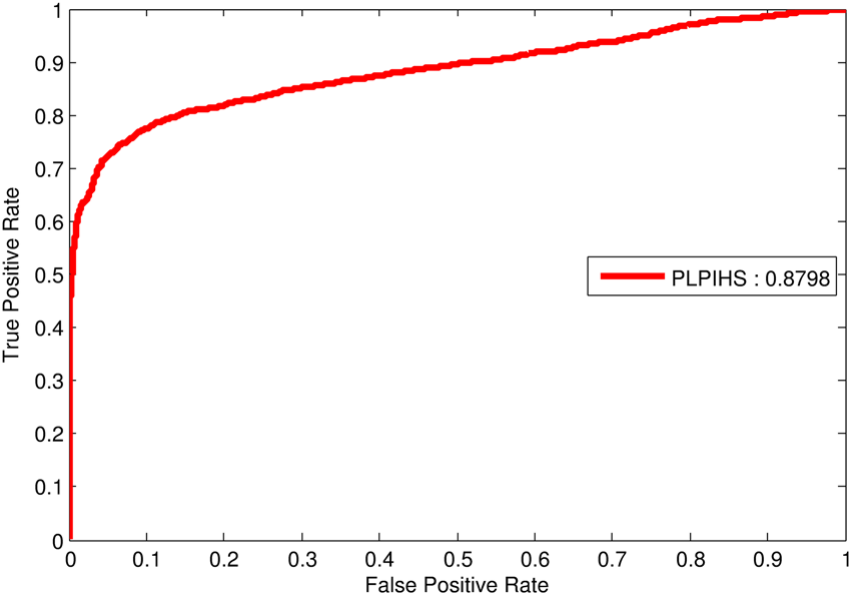
The ROC curves of the independent test set in PLPIHS.

For further validation, we also randomly selected 2000 lncRNA-protein associations from the rest of positive samples in 0.9 network and the same number of negative interactions are picked out from the remaining negative samples of 0.3 network to generate the independent test data set. Since the existing network based methods is not suitable for independent test, we only evaluate the performance for the proposed PLPIHS. The independent test results are shown in Fig. 6, an AUC score of 0.879 is achieved by PLPIHS, illustrating the effectiveness and advantage of the proposed approach.

### Case Studies

By applying the proposed PLPIHS method, novel candidate lncRNA-related proteins are predicted using LOOCV. We applied PLPIHS onto the 2000 known lncRNA-protein associations, which includes 1511 lncRNAs and 344 proteins to infer novel lncRNA-protein interactions. As a result, an area under the ROC curve of 0.9669, 0.9705 and 0.9703 (Fig. 5) is achieved using the three networks of different connectivity density, which demonstrate that our proposed method is effective in recovering known lncRNA-related proteins.

To further illustrate the application of our approach, a case study of lncRNA MALAT1(ensemble ID: ENSG00000251562) is examined. MALAT1 is a long non-coding RNA which is over-expressed in many human oncogenic tissues and regulates cell cycle and survival^31^. MALAT1 have been identified in multiple types of physiological processes, such as alternative splicing, nuclear organization, epigenetic modulating of gene expression. A large amount of evidence indicates that MALAT1 also closely relates to various pathological processes, including diabetes complications, cancers and so on^32, 33^.

**Figure 7:**
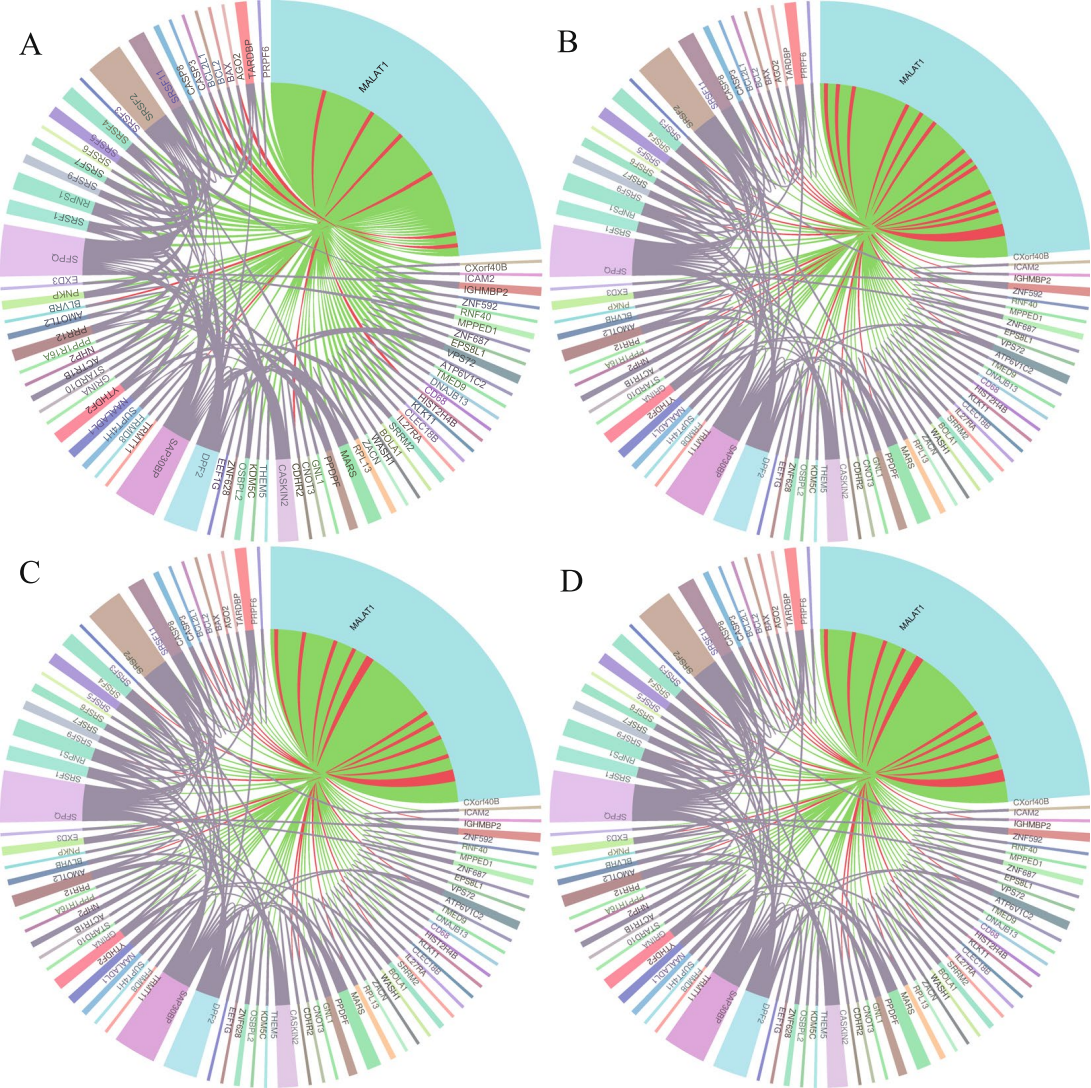
Case study of lncRNA MALAT1. The four chord graphs show the results of PLPIHS (**A**), PRINCE (**B**), RWR (**C**) and LPIHN (**D**) respectively. The biggest baby blue node represents lncRNA MALAT1 and the other nodes are related proteins, red lines denotes wrong prediction while green lines are correct prediction and the violet lines means the interactions between proteins.

MALAT1 is associated with 68 proteins in NPInter 3.0^34^. We construct the interaction networks of lncRNA MALAT1 by using the prediction results of these four methods (Fig. 7). Among the 68 known lncRNA-protein interactions, PLPIHS wrongly predicts 6 interactions, while 13 associations are predicted mistakenly by PRINCE and RWR method and 15 lncRNA-protein pairs are falsely predicted by the LPIHN method.

We manually check the top 10 proteins in the ranked list under 0.5 network (Table 3).Three of the top 10 predicted proteins have interactions with MALAT1, and most of them had high ranks in the predicted protein lists. For example, In the investigation of colorectal cancer (CRC), MALAT1 could bind to SFPQ, thus releasing PTBP2 from the SFPQ/PTBP2 complex and the interaction between MALAT1 and SFPQ could be a novel therapeutic target for CRC^35^. MALAT1 interacts with SR proteins (SRSF1, SRSF2, SRSF3 and SRSF5) and regulates cellular levels of phosphorylated forms of SR proteins^36^. And it is also as target of TARDBP to play the biological performance and found that TDP-43 bound to long ncRNAs in highly sequence-specific manner in tissue from subjects with or without FTLD-TDP, the MALAT1 ncRNA recruits splicing factors to nuclear speckles and affects phosphorylation of serine/arginine-rich splicing factor proteins^37, 38^. All these results indicate that our proposed method is effective and reliable in identifying novel lncRNA-related proteins.

**Table 3:** The top 10 ranked proteins for lncRNA MALAT1(ensemble ID: ENSG00000251562).

protein	ensemble ID	rank
SFPQ	ENSG00000116560	1
SRSF5	ENSG00000100650	2
SRSF11	ENSG00000116754	3
MARS	ENSG00000166986	4
CASP8	ENSG00000064012	5
TARDBP	ENSG00000120948	6
IGHMBP2	ENSG00000132740	7
SRSF7	ENSG00000115875	8
PRR12	ENSG00000126464	9
SRSF2	ENSG00000161547	10

## Discussion

LncRNAs are involved in a wide range of biological functions through diverse molecular mechanisms often including the interaction with one or more protein partners^12, 13^. Only a small number of lncRNA-protein interactions have been well-characterized. Computational methods can be helpful in suggesting potential interactions for possible experimentation^25^. In this study, we use HeteSim measure to calculate the relevance between lncRNA and protein in a heterogeneous network. The importance of inferring novel lncRNA-protein interactions by considering the subtle semantic meanings of different paths in the heterogeneous network have been verified^39^. We first construct a heterogeneous network consisting of a lncRNA-lncRNA similarity network, a lncRNA-protein association network and a protein-protein interaction network. Then, we use the HeteSim measure to calculate a score for each lncRNA-protein pairs in each path. Finally, a SVM classifier is used to combine the scores of different paths and making predictions. We compare the proposed PLPIHS with PRINCE, RWR and LPIHN and find that PLPIHS obtain an AUC score of 0.9679 in 0.3 network, which is significantly higher than PRINCE, RWR and LPIHN (0.813, 0.884 and 0.7918, respectively). We also compare the performance of these four methods in networks of different connectivity density. As a result, PLPIHS outperforms the other method across all the networks. Moreover, when analysing the predicted proteins interacted with lncRNA MALAT1, PLPIHS successfully predicts 63 out of 68 associations, while PRINCE, RWR and LPIHN retrieve much lower interactions of 57, 57 and 53, respectively. And the top-ranked lncRNA-protein interactions predicted by our method are supported by existing literatures. The results highlight the advantages of our proposed method in predicting possible lncRNA-protein interactions.

## Methods

### lncRNA–Protein associations

All human lncRNA genes and protein-coding genes are downloaded from the GENCODE Release 24^9^. A total of 15941 lncRNA genes and 20284 protein-coding genes are extracted. To obtain genome-wide lncRNA and protein-coding gene associations, we combine three sources of data:

- Co-expression data from COXPRESdb^40^. Three preprocessed co-expression datasets (Hsa.c4-1, Hsa2.c2-0 and Hsa3.c1-0) including pre-calculated pairwise Pearson’s correlation coefficients for human were collected from COXPRESdb. The correlations are calculated as follows:
  1 $$C(l,p)=1-\prod _{d=1}^{D}\mathrm{(1}-{C}_{d}(l,p))\quad if{C}_{d}(l,p) > 0$$
  where *C*(*l*, *p*) is the overall correlation between gene *l* (lncRNA) and protein-coding gene *p*, *C*_*d*_(*l*, *p*) is the correlation score between *l* and *p* in dataset *d*, *D* is the number of gene pairs (*l* and *p*) with positive correlation scores. Gene pairs with negative correlation scores are removed.
- Co-expression data from ArrayExpress^41^ and GEO^42^. We obtained the co-expresionn data from the work of Jiang *et al*.^43^. RNA-Seq raw data of 19 human normal tissues are obtained from ArrayExpress (E-MTAB-513) and GEO (GSE30554). TopHat and Cufflinks with the default parameters are used to calculate the expression values. Pearson’s correlation coefficients are used to evaluate the co-expression of lncRNA-protein pairs.
- lncRNA-protein interaction data. We download known lncRNA-protein interaction dataset from Npinter 3.0^34^ in April 2016 and then filter the lncRNAs and their interaction proteins, by restricting the organism of lncRNAs to “Homo sapiens”. The score *I*(*l*, *p*) is 1 if there is an interaction between lncRNA *l* and protein *p*, otherwise the score is 0.

### LncRNA co-expression similarity

The lncRNA expression profiles are obtained from NONCODE2016 database^44^ (downloaded on April 6, 2016), including the expression profiles of 90,062 lncRNA in 24 human tissues or cell types. Pearson’s correlation coefficient between the expression profiles of each pair of lncRNAs is calculated as the similarity.

### Protein-protein interactions

We obtain the protein-protein interaction (PPI) data from STRING database V10.0^45^, which contains weighted protein interactions derived from computational prediction methods, high-throughput experiments, and text mining. The confidence scores are computed by combining the probabilities from the different evidence channels, correcting for the probability of randomly observing an interaction.

### The HeteSim measure

The HeteSim measure is a uniform and symmetric relevance measure. It can be used to calculate the relatedness of objects with the same or different types in a uniform framework, and it is also a path-constrained measure to estimate the relatedness of object pairs based on the search path that connects two objects through a sequence of node types^39^. Further, the HeteSim score has some good properties (i.e., selfmaximum and symmetric), which have achieved positive performance in many studies^25^. In this study, we use HeteSim scores to measure the similarities between lncRNAs and proteins.

**Definition 1** Transition probability matrix^39^ L and P are two kinds of object in the heterogeneous network, (*I*_*LP*_)_n*m_ is an adjacent matrix between *L* and *P*, then the normalized matrix of *I*_*LP*_ along the row vector is defined as

2 $${T}_{LP}(i,j)=\frac{{I}_{LP}(i,j)}{{\sum }_{k=1}^{m}{I}_{LP}(i,k)}$$

**Definition 2** Reachable probability matrix^39^ In a heterogeneous network, the reachable probability matrix ${R}_{{\mathscr{P}}}$ for path ${\mathscr{P}}=({P}_{1}{P}_{2}\cdots {P}_{n+1})$ of length n, where *P*_*i*_ belongs to any objects in the heterogeneous network, can be expressed as

3 $${R}_{{\mathscr{P}}}={T}_{{P}_{1}{P}_{2}}{T}_{{P}_{2}{P}_{3}}\cdots {T}_{{P}_{n}{P}_{n+1}}$$

**Figure 8:**
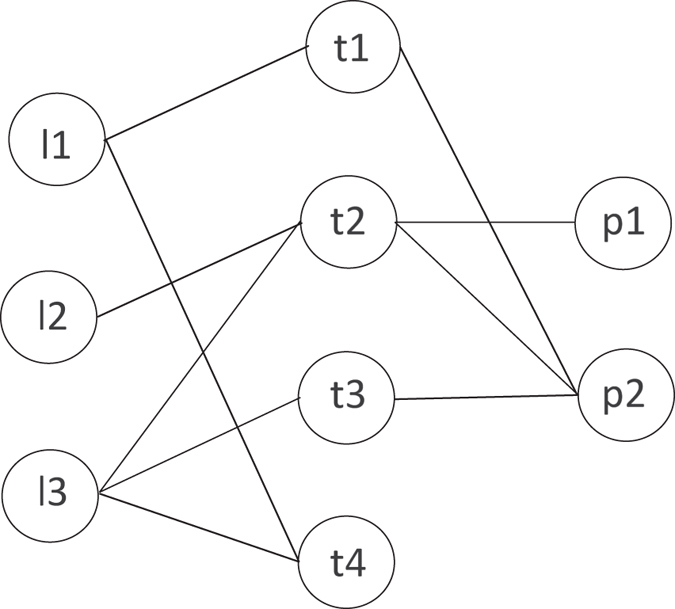
Example of calculating HeteSim score.
- Split the path ${\mathscr{P}}$ into two components ${{\mathscr{P}}}_{L}=(LT)$ and ${{\mathscr{P}}}_{R}=(TP)$.
- Given the adjacent matrix *I*_*LT*_ and *I*_*TP*_ below, which means the interactions between lncRNAs and proteins, we can obtain the transition probability matrix *T*_*LT*_ and *T*_*TP*_ by normalizing the two matrix along the row vector. Actually, the reachable probability matrix for path ${{\mathscr{P}}}_{L}$ and ${{\mathscr{P}}}_{R}$ are equivalent their corresponding transition probability matrix, for example, ${R}_{{{\mathscr{P}}}_{L}}$ = *T*_*LT*_ and ${R}_{{{\mathscr{P}}}_{L}}$ = *T*_*TP*_.
  $$\begin{array}{rcl}{I}_{LT} & = & [\begin{array}{cccc}1 & 0 & 0 & 1\\ 0 & 1 & 0 & 0\\ 0 & 1 & 1 & 1\end{array}]\,{I}_{PT}=[\begin{array}{cccc}0 & 1 & 0 & 0\\ 1 & 1 & 1 & 0\end{array}]\\ {T}_{LT} & = & [\begin{array}{cccc}0.5 & 0 & 0 & 0.5\\ 0 & 1 & 0 & 0\\ 0 & 0.33 & 0.33 & 0.33\end{array}]\,{T}_{PT}=[\begin{array}{cccc}0 & 1 & 0 & 0\\ 0.33 & 0.33 & 0.33 & 0\end{array}]\end{array}$$
- Calculate the HeteSim score for each pair in the network. and the hetesim score matrix are displayed below. i.e.,

Based on the definitions above, the steps of calculating HeteSim scores between two kinds of objects (lncRNA and protein) can be presented as follows:

- Split the path into two parts. When the length n of path ${\mathscr{P}}$ is even, we can split it into ${{\mathscr{P}}}_{L}=({P}_{1}\cdots {P}_{mid})$ and ${{\mathscr{P}}}_{R}=({P}_{mid}\cdots {P}_{n+1})$, where $mid=(\frac{n}{2})+1$ Otherwise, if n is odd, the path cannot be divided into two equal-length paths. In order to deal with such problem, we need to split the path twice by setting $mid=\frac{(n+\mathrm{1)}}{2}$ and $mid=\frac{(n+3)}{2}$, respectively. Then, we can obtain a HeteSim score for each mid value, the final score will be the average of the two scores.
- Achieve the transition probability matrix and reachable probability matrix under the path ${{\mathscr{P}}}_{L}$ and ${{\mathscr{P}}}_{R}$.
- Calculate the HeteSim score:
  4 $$HeteSim(l,p|{\mathscr{P}})=\frac{{R}_{{{\mathscr{P}}}_{L}}(l,\,:){({R}_{{{\mathscr{P}}}_{R}^{-1}}(p,:))}^{T}}{{\Vert {R}_{{{\mathscr{P}}}_{L}}(l,:)\Vert }_{2}\times {\Vert {R}_{{{\mathscr{P}}}_{R}^{-1}}(p,:)\Vert }_{2}}$$
  where ${{\mathscr{P}}}_{R}^{-1}$ is the reverse path of ${{\mathscr{P}}}_{R}$.
  An example of calculating HeteSim score is indicated in Fig. 8. We can see that there are three kinds of objects L, T and P in the network. The simplified steps of computing HeteSim score between l3 and p2 under the path ${\mathscr{P}}$ = (LTP) is as follows:

$$HeteSim(l3,p1|{\mathscr{P}})={I}_{LT}(3,\,:){I}_{TP}(:,2)=0.5774$$

$$[\begin{array}{ccc} & p1 & p2\\ l1 & 0 & 0.4082\\ l2 & 1 & 0.5774\\ l3 & 0.5774 & 0.6667\end{array}]$$

### The PLPIHS method

Among a heterogeneous network, different paths can express different semantic meanings. For instance, a lncRNA and a protein is connected via ‘lncRNA-lncRNA-protein’ path or ‘lncRNA-protein-protein’ path representing totally different meanings. The former means that if a lncRNA is associated with a protein, then another lncRNA similar to the lncRNA will be potential associated with the protein. The latter shows that if a protein associated with a lncRNA, then another protein interacted with the protein will be likely associated with the lncRNA. Therefore, the potential information hidden in each path is extraordinary essential to be taken into account during prediction.

The PLPIHS framework is illustrated in Fig. 2. Firstly, we construct a heterogeneous network consisting of a lncRNA-lncRNA similarity network, a lncRNA-protein association network and a protein-protein interaction network. Three kinds of sparse networks are obtained from the heterogeneous network under different cutoff value 0.3, 0.5 and 0.9 (see lncRNA-Protein associations). The larger cutoff is, the network is more sparse. A total of 15941 lncRNAs genes and 20284 protein-coding genes are extracted as presented in Section 2.3. We randomly take out 1511 lncRNAs and 344 proteins to construct a smaller network for the following experiments in consideration of computing costs. The construction of the smaller heterogeneous networks under different cutoff values are shown in Table 4, where ‘lnc2lnc’ denotes the lncRNA-lncRNA network, ‘lnc2code’ denotes the lncRNA-protein network and ‘code2code’ denotes the protein-lncRNA network. For example, there are 25,469 interactions in the lncRNA-protein network under the cutoff if 0.3. Then, we randomly select 2000 lncRNA-protein pairs as positive examples from the 0.9 network and the same number of interaction pairs as negative examples from the 0.3 network. The paths with length less than six transferred from a lncRNA to a protein among the heterogeneous network are listed in Table 1. We use id to indicate the path combination, i.e., 1′~2′ represents path ‘LLP’ and path ‘LPP’. Next, we calculate the heteSim score for each lncRNA-protein pair under each path. The results of different paths are used as different features. And we combine a constant factor *β* to inhibit the influence of longer paths.The longer the path length is, the smaller the inhibiting factor is. Finally, a SVM classifier is built with these scores to predict potential lncRNA-protein associations. On the account of the HeteSim measure is based on the path-based relevance framework^39^, it can effectively dig out the subtle semantics of each paths.

**Table 4:** Construction of the three networks under different cutoffs.

network	lnc2lnc	lnc2code	code2code
0.3	61,469	25,469	8,362
0.5	34,849	14,700	5,390
0.9	7,799	9,086	3,180
